# Polytetrafluoroethylene tape spacer for direct splinted teeth-supported provisional restoration: A dental technique

**DOI:** 10.1016/j.heliyon.2023.e15035

**Published:** 2023-04-05

**Authors:** Hayam Alfallaj, Faris Z. Jamjoom, Abdulaziz A. Alzaid, Hatem Alqarni

**Affiliations:** a Restorative and Prosthetic Dental Sciences Department, College of Dentistry, King Saud Bin Abdulaziz University for Health Sciences, Riyadh, Saudi Arabia; b King Abdullah International Medical Research Center, Ministry of National Guard for Health Affairs, Riyadh, Saudi Arabia

**Keywords:** PTFE, Bis acryl, Provisional, Restoration, Direct splin

## Abstract

Provisional restoration is essential for function, esthetics, occlusion, and tissue health. However, when multiple teeth are involved, making a direct splinted provisional restoration could be challenging due to the combined undercuts between the prepared teeth. This article describes a dental technique using polytetrafluoroethylene (PTFE) tape as a spacer to fabricate a splinted provisional restoration for multiple prepared teeth. The PTFE tape spacer will compensate for discrepancies in the path of insertion between prepared teeth, thus facilitating the removal of the direct splinted provisional restoration intact.

## 1 Introduction

Provisional restorations following tooth preparation can serve several functions until the final restorations are ready for cementation. These include covering exposed dentine to prevent sensitivity, prevent unwanted proximal and occlusal tooth movement, facilitate proper oral hygiene, prevent gingival overgrowth, and provide adequate esthetics and function. In addition, provisional restorations can serve as a diagnostic aid to assess patient acceptance and tolerance of esthetic and occlusal changes [1,2].

Direct or chair-side provisional restorations can be fabricated out of an appropriate provisional materials using matrices made of impression materials or vacuum-formed celluloid acetate material that closely replicates teeth morphology before preparation or the desired morphology based on a diagnostic wax pattern. Direct provisional restorations are both time and cost-efficient as they eliminate laboratory time and expenses required for fabricating indirect lab-processed provisional restorations [1,2]. Some procedural difficulties may be encountered when fabricating direct provisional restorations. Failure to account for the cement can produce seating errors which may lead to open margins and premature occlusal contacts [1,2]. Furthermore, without adequate separating materials, certain provisional materials such as Bis-GMA could bond to composite resin restoration or core of the prepared tooth preventing removal of the provisional restoration intact and risking damage to the composite resin restoration [3,4]. Direct provisional restorations can be particularly challenging when multiple adjacent teeth are prepared. Individually made provisional crowns present considerable difficulties when optimizing proximal and occlusal contacts [1,2]. Therefore, it has been a common practice to splint adjacent provisional crowns into a single unit to overcome problems related to teeth contact, thus eliminating the risk of teeth drifting [1,2]. Additionally, splinted provisional restorations are more structurally durable and less likely to debond than un-splinted individual crowns [1,2].

The initial removal of splinted direct provisional restorations to trim the excess material and open the embrasures might be difficult, especially when the prepared teeth have discrepancies in their paths of withdrawal. In this situation, the combined proximal surfaces of the prepared teeth create undercuts that are engaged by the provisional material, potentially locking the restoration in place [5].

Polytetrafluoroethylene (PTFE) tape, commonly known as plumber's or Teflon tape, has been commonly used in dental procedures, such as sealing the coronal aspect of endodontic fillings and screw access channel in implant-supported restorations [6,7]. PTFE tape has also been used to simplify the removal of a well-fitted onlay/inlay restoration during the try-in stage, to block-out unfavorable undercuts or deep embrasures during impression making, to simplify the repair of a fractured abutment tooth under pre-existing crown, to prevent subgingival stagnation of excess cement around a tooth and implant-supported restorations, and as a barrier to prevent etching and bonding of the interproximal area of adjacent teeth [6,[8], [9], [10], [11], [12]].

In this article, a simple technique for multiple adjacent prepared teeth with different insertion paths is described. In which, PTFE tape is used as a spacer between prepared teeth and direct provisional restoration. This procedure ease the removal of the provisional restoration from the patient mouth for further shaping and contouring. Also, it creates a cement space reducing seating errors and open margins, allowing better maintenance of the provisional restoration and the periodontal health until cementation of the final crowns.

## 2 Technique

**Fig. 1:**
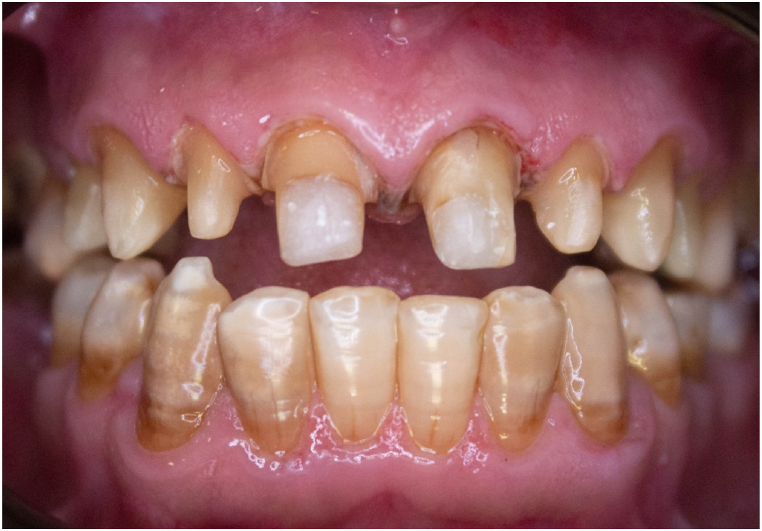
Frontal view of the preparation ready for provisional restorations.

1. Fabricate a silicone putty matrix index (Aquasil Soft Putty Impression Material; Dentsply) on the pre-teeth preparation diagnostic cast or the diagnostic wax pattern cast.
2. Use a scalpel to cut the putty matrix index extensions beyond the teeth gingival margins thereby opening the embrasures. This will facilitate removal of excess provisional material.
3. Cut strips of PTFE tape for each prepared tooth. The length of each strip depends on the size of the prepared tooth. One layer per tooth is usually enough. However, when larger discrepancies in the parallelism between the prepared teeth are present, additional PTFE tape layers can be used to block combined undercuts between the prepared teeth.
4. Use a plastic instrument (Universal thin PFI; Hufriedy Group) to closely adapt the PTFE tape around all surfaces of the prepared teeth. To overcome marginal misfit, keep the PTFE tape 1 mm coronal to the preparation finish line (Fig. 1, Fig. 2).

**Fig. 2:**
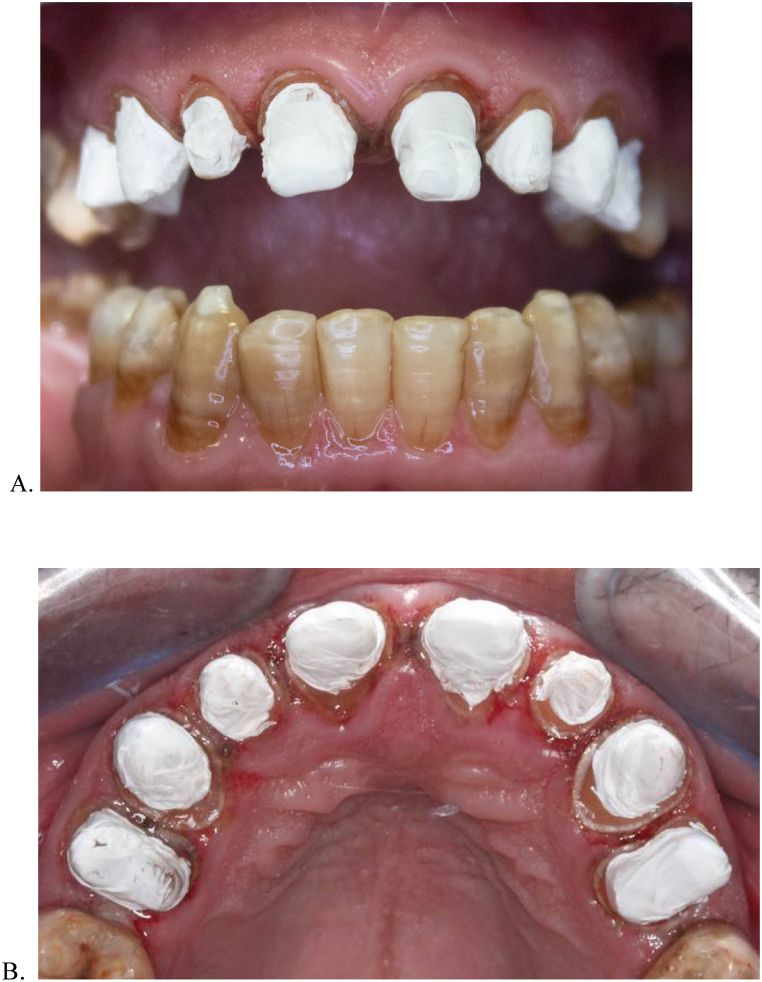
A, Frontal view of the (PTFE) barrier placed 1-mm away from the preparation margin. B, Occlusal View.
5. Dispense Bis-Acryl provisional material (Protemp Plus temporization Material; 3 M ESPE) according to the manufacturer's instructions into the putty matrix index.
6. Insert the putty matrix index intra-orally and ensure full seating. The occlusal surface of unprepared teeth can be used as a guide to verify seating putty matrix index in its correct position.
7. Remove the excess Bis-acryl provisional material that extrudes from the putty matrix index opened embrasures.
8. Once the provisional material has set according to the manufacturer-reported setting time, remove the putty matrix index. As the PTFE will act as a spacer, easier removal of the splinted provisional restoration is anticipated (Fig. 3).

**Fig. 3:**
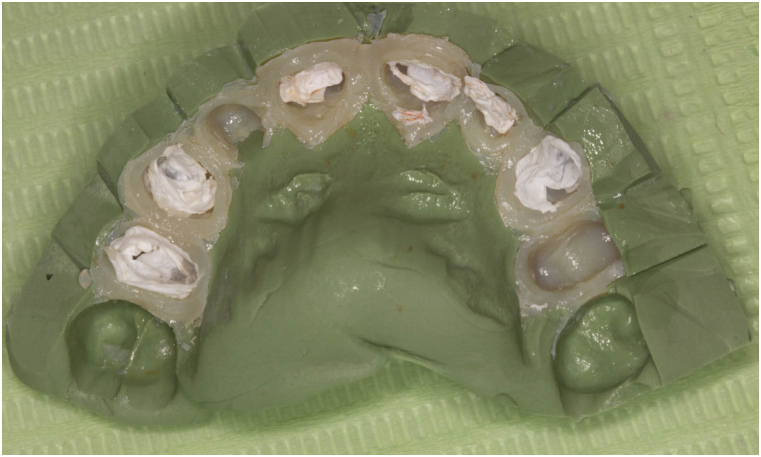
Occlusal view of provisional restoration showing the residual (PTFE).
9. Dry the provisional restoration and remove any attached Teflon tape from the intaglio surface of the provisional restoration using cotton pliers or a hemostat. Firmly attached Teflon tape may require removal using acrylic bur.
10. Asses the resulting provisional restoration for the presence of any voids. Use flowable composite (Filtek Supreme Flowable Restorative; 3 M ESPE) according to the manufacturer to fill any voids.
11. Finish the margins and open the embrasures using discs and finishing burs (Fig. 4A).

**Fig. 4:**
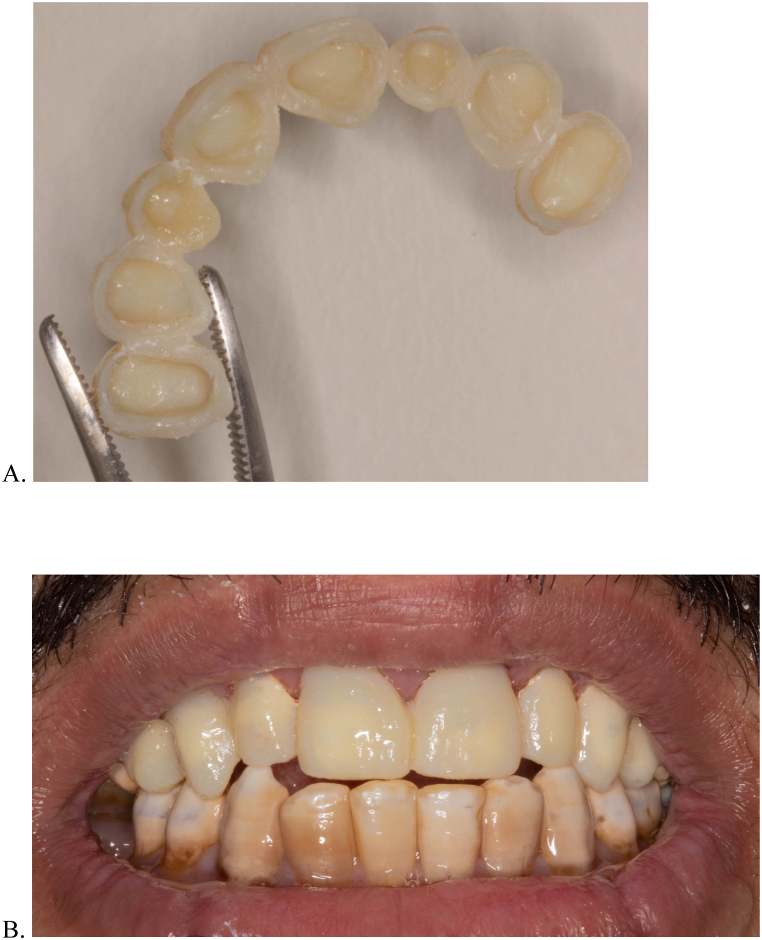
A, Extraoral occlusal view of the provisional restoration after adjustments. B, Intraoral frontal view of the provisional restoration.
12. Confirm the seating of the provisional restoration, verify adequate marginal integrity, and check occlusion using articulating paper and adjust using acrylic bur accordingly.
13. Use provisional cement (RelyX Temp NE; 3 M ESPE) to cement the provisional restoration. After setting, carefully remove excess cement and check occlusion using articulating paper (Fig. 4B).

### 2.1 Summary

The technique presented in this article facilitates chair-side fabrication of direct splinted provisional restoration for multiple teeth. Adjacent teeth prepared for individual crowns can have discrepancies in their insertion path which makes fabrication of a splinted provisional restoration challenging. The PTFE tape may compensate for such discrepancies reducing the likelihood of locking or breakage during removal, thereby facilitating fabrication of single provisional restoration for multiple prepared teeth with different insertion paths.

## Author contribution statement

1 - Conceived and designed the experiments: Hayam Alfallaj, and Hatem Alqarni.

2 - Performed the experiments: Hayam Alfallaj, and Hatem Alqarni

3 - Analyzed and interpreted the data: Hayam Alfallaj, Faris Jamjoom, Abdulaziz Alzaid, and Hatem Aqarni.

4 - Contributed reagents, materials, analysis tools or data: Hayam Alfallaj, Faris Jamjoom, Abdulaziz Alzaid, and Hatem Aqarni.

## Funding statement

This research did not receive any specific grant from funding agencies in the public, commercial, or not-for-profit sectors.

## Data availability statement

No data was used for the research described in the article.

## Declaration of interest's statement

The authors declare no conflict of interest.

## Additional information

No additional information is available for this paper.
